# A qualitative metasummary of detransition experiences with recommendations for psychological support

**DOI:** 10.1016/j.ijchp.2024.100467

**Published:** 2024-05-07

**Authors:** Pablo Expósito-Campos, José Ignacio Pérez-Fernández, Karmele Salaberria

**Affiliations:** a Department of Clinical and Health Psychology and Research Methods, Faculty of Psychology, University of the Basque Country - Tolosa Hiribidea 70, 20018 Donostia-San Sebastián, Gipuzkoa, Spain; b Predoctoral Research Fellowship Program of the Department of Education of the Government of the Basque Country, Spain

**Keywords:** Gender transition, Gender detransition, Qualitative metasummary, Psychological support

## Abstract

**Objective:**

The main goal of this article is to identify areas of psychotherapeutic work with detransitioners, that is, individuals who stop or reverse a gender transition, given the scarcity of information and resources.

**Methods:**

We conducted a systematic review and metasummary of qualitative data published until April 2023. Data were extracted, grouped, and refined to conform meta-findings.

**Results:**

The database search yielded 845 records, of which 15 comprising 2689 people who detransitioned were included in the review. A total of 582 findings were extracted, resulting in 34 meta-findings with frequencies ≥ 15 %. Two main thematic areas with several subthemes were identified. The theme “Gender transition” included “Perspectives” and “Emotions.” The theme “Gender detransition” included “Driving factors,” “Challenges” (a. Social and emotional difficulties, b. Lack of support and understanding, c. Negative healthcare experiences, d. Detransphobia, and e. Identity concerns), “Needs,” “Growth and evolution,” and “Identity and future.” Based on these meta-findings, we advance broad recommendations for supporting detransitioners in their various emotional, social, and identity needs.

**Conclusions:**

Detransitioners are diverse in their experiences and perspectives and face significant challenges. Emotional validation with a focus on personal strengths and meanings, treatment of concurrent psychological issues, development of social networks, and support of identity exploration are key aspects of psychotherapeutic work with this population.

## Introduction

The experiences of transgender and gender diverse (TGD) people, that is, those whose gender identities differ from their natal sex, have received significant interest and attention over the last decade. This has fueled the development and expansion of comprehensive centralized and decentralized care services (Koehler et al., 2021) and guidelines (Coleman et al., 2022) that seek to meet the needs of a growing population of TGD individuals (Zhang et al., 2020), which may involve gender affirmation through social (e.g., changing name and pronouns) or medical (e.g., gender-affirming hormone therapy) means. However, recent academic and media articles have turned their focus to individuals who *detransition*, that is, who stop or reverse their gender transition, who may self-label as *detransitioners* or *detrans*. Often, this is associated with a shift in identity and may include steps such as reverting to a previous gender expression or discontinuing gender-affirming medical treatment (Littman, 2021; MacKinnon, et al., 2022b; Pullen Sansfaçon, Gelly, et al., 2023; Vandenbussche, 2022). The prevalence of detransition fluctuates between less than 1 % and over 13 %, although estimates vary considerably according to case definitions and are affected by conceptual and methodological shortcomings (Expósito-Campos et al., 2023). The last decade has seen an increase in the number of young people transitioning and detransitioning, leading to controversy about possible driving factors, particularly changing models of care, unrecognized psychological problems, and peer influence (Jorgensen, 2023).

Cross-sectional online surveys of clinicians working in TGD healthcare (Narayan et al., 2021; Pullen Sansfaçon, Planchat, et al., 2023) and retrospective case note reviews from gender identity services (Butler et al., 2022; Hall et al., 2021; Pazos Guerra et al., 2020) provide emerging evidence of detransition-related clinical encounters. Despite this, there is currently no guidance to support providers working with individuals who detransition (Jorgensen, 2023; MacKinnon et al., 2022b) and informal resources are scarce. In practice, this means that individuals who detransition may have unmet health needs (MacKinnon et al., 2023), while providers may be unaware of or unprepared to respond to the diversity of detransition experiences and trajectories (Jorgensen, 2023). This has prompted numerous calls for rigorous research and the development of detransition-centered care services (Butler & Hutchinson, 2020; Entwistle, 2021; Irwig, 2022; MacKinnon et al., 2023).

The current review aims to partially address this gap by identifying the most relevant areas of intervention and advancing broad psychotherapeutic recommendations for working with people who detransition. More specifically, we aim to answer the following questions: (1) What are the psychosocial experiences and challenges faced by people who detransition?; and (2) What can psychologists do to promote their well-being? Given the proposed aim, the focus of the review will be on qualitative data, as it has the potential to deepen and provide richer and more detailed information about detransition experiences. To our knowledge, there have been no previous attempts to conduct a synthesis of existing qualitative evidence to help mental health providers gain a comprehensive understanding of the experiences and needs of detransitioners.

## Methods

### Design

We conducted a *qualitative metasummary* following Sandelowski and Barroso (2007), which is a quantitatively oriented aggregation of qualitative data through the identification of recurring findings or themes. The results of this qualitative synthesis were reported in accordance with the *Enhancing transparency in reporting the synthesis of qualitative research* (ENTREQ) statement (Tong et al., 2012). The protocol for the review can be found in the *International prospective register of systematic reviews* (PROSPERO) with the code CRD42023413888.

### Search strategy

A systematic literature search was conducted in four databases (PubMed, Scopus, Web of Science Core Collection, and APA PsycINFO) using the strategy shown in Table S1 in the Supplementary Materials. Search terms were applied to the Title, Abstract, and Keywords fields, with adjustments made to the advanced search features of each specific database. We also conducted a hand search of the references of studies assessed for eligibility to identify additional records missed by the electronic search. Finally, other relevant studies known to the authors were included to ensure the comprehensiveness of the review.

### Eligibility criteria

Eligibility criteria for studies included in the review were determined using the *Sample, Phenomenon of interest, Design, Evaluation, and Research type* (SPIDER) tool (Cooke et al., 2012). To be included, studies had to meet the following criteria: (a) use the experiences of people who detransition as their primary source of data; (b) present a qualitative or mixed methods research design, with qualitative data distinguishable from other types of data; and (c) be published in English or Spanish from database inception until April 2023. Dissertations and book chapters were considered eligible due to their potential relevance to the review.

The following records were excluded: (a) studies with quantitative or mixed methods designs without separate presentation or analysis of qualitative data; (b) theoretical and opinion articles without primary qualitative data; and (c) systematic and literature reviews, conference papers, abstracts, and other types of grey literature. Autobiographies and first-person accounts were also excluded because their analysis would have required a different methodological approach.

We initially considered excluding studies that mixed data from detransitioners and other populations. However, anticipating that only a small number of studies would meet this criterion, we ultimately decided to extend the scope of the review to include any study with detransitioners among their participants. Full eligibility criteria can be found in Table S2 in the Supplementary Materials.

After removing duplicates, the eligibility of each record was determined based on the title and abstract. The full text was retrieved only when the title and abstract alone were insufficient to assess eligibility. Potentially eligible records were retrieved and assessed before final selection and inclusion in the qualitative metasummary. Each record was assessed independently by two authors (PEC and JIPF) and discrepancies were resolved by a third author (KS). The process was conducted according to the *Preferred Reporting Items for Systematic Reviews and Meta-Analyses* (PRISMA) declaration (Page et al., 2021).

### Data extraction

The following information was extracted from each record: (a) author, year, country, and type of publication; (b) aim of the study; (c) sample characteristics; (d) methodology; and (e) type of finding. To determine (e), we followed the typology of qualitative findings proposed by Sandelowski and Barroso (2007), which considers the extent of data interpretation by the authors of each study (Table S3 in the Supplementary Materials).

### Methodological quality

The Mixed Methods Appraisal Tool (MMAT) version 2018 (Hong et al., 2019) was used to assess the methodological quality of the studies included in the review. It consists of two screening items and five items specific to each study category. The response options are “Yes,” “No,” and “Can't tell,” so a higher number of items answered with “Yes” reflects higher methodological quality and a lower risk of bias, and vice versa. For the current review, we only used the criteria for the categories of qualitative and mixed-methods studies. Following Sandelowski and Barroso (2007), the methodological quality of the studies was not used as a criterion for inclusion or exclusion in the metasummary.

### Data synthesis and analysis

We followed Sandelowski and Barroso's (2007) five-step approach to conducting qualitative metasummaries. First, we extracted findings from each study that addressed the research questions. For this purpose, we defined *detransition-related findings* as any researcher interpretation that addressed the experience of detransition, including: (a) factors influencing the decision to detransition; (b) the emotional and social experiences associated with detransition; (c) facilitators and barriers to seeking professional support; and (d) detransitioners’ reflections on their gender process. We considered experiences of detransition that involved stopping or reversing any of the social, medical, or legal changes made during gender transition. Second, the extracted findings were edited to improve their clarity, while retaining their original structure and language as far as possible. Third, the findings were grouped into broad categories based on thematic similarity. Fourth, the findings were reduced and refined until we had a set of findings (*meta-findings*) that conveyed the essence of all the original findings. Finally, we calculated frequencies by dividing the number of studies containing each meta-finding by the total number of included studies. We also calculated the contribution of studies by dividing the number of meta-findings present in each study by the total number of meta-findings. Only meta-findings with frequencies ≥ 15 % were included.

## Results

**Fig. 1:**
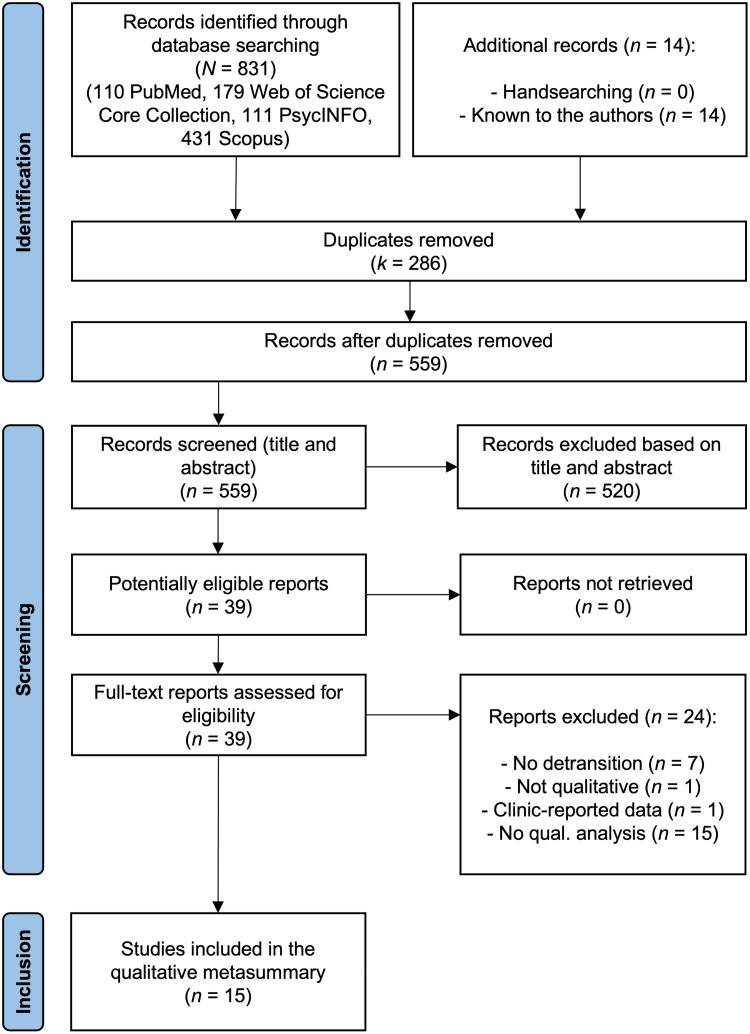
PRISMA flow chart.

The search yielded a total of 845 records, of which 559 were unique after removal of duplicates (*k* = 286). The process of screening against the inclusion and exclusion criteria resulted in the final selection of 15 studies for inclusion in the qualitative metasummary (Fig. 1), with an inter-rater agreement of 89 %. The main characteristics of each study are shown in Table 1.

**Table 1:** Characteristics of included studies.

**ID number, author (year), country, type of publication**	**Aim**	**Sample characteristics**	**Methodology**	**Type of finding**
1, Cain and Velasco (2021), US, journal article	Explore the intersection between gender identity, sexuality, and disability	*N* = 1 (1 F) Age NS 1 med	Semi-structured interview, thematic analysis	Topical survey
2, *Durwood et al., 2022, US, journal article	Explore factors, reactions, and reflections related to detransition	*N* = 31 (10 detrans, 21 parents) Natal sex and age NS 10 soc	Semi-structured interview, thematic analysis	Thematic survey
3, Haarer (2022), US, dissertation	Determine the essence of detransition	*N* = 10 (9 F, 1 M) Age range = 19–59 3 soc, 7 med	Semi-structured interview, phenomenological analysis	Thematic survey
4, Kuiper and Cohen-Kettenis (1998), Netherlands, journal article	Study regret following surgical transition	*N* = 10 (9 M, 1 F) *M* age = 46 10 med	Semi-structured interview,^b^ analysis NS	Topical survey
5, Littman (2021), US, journal article	Describe detransition reasons and narratives	*N* = 100 (69 F, 31 M) *M* age = 29.2 100 med	Survey, thematic analysis^b^	Thematic survey
6, MacKinnon, Gould, et al. (2022a), Canada, journal article^a^	Conceptualize detransphobia as a minority stressor	*N* = 28 (18 F, 10 M) Age range = 20–53 1 soc, 27 med	Semi-structured interview, comparative analysis	Conceptual description
7, MacKinnon, Kia, et al. (2022b), Canada, journal article^a^	Explore physical and mental health among detransitioners	*N* = 28 (18 F, 10 M) Age range = 20–53 1 soc, 27 med	Semi-structured interview, comparative analysis	Topical survey
8, Pullen Sansfaçon, Gelly, et al. (2023), Canada, journal article	Explore experiences and feelings about transition and detransition	*N* = 20 (19 F, 1 M) *M* age = 21.3 7 soc, 13 med	Semi-structured interview, thematic analysis	Thematic survey
9, Sanders et al. (2023), US, journal article	Explore detransition narratives on social media	*N* = 36 (19 detrans) Natal sex, age, and transition NS	Sampling of social media posts, thematic analysis	Thematic survey
10, Shepherd & Hanckel (2021), Australia, journal article	Explore transition in healthcare documents vs. people's experiences	*N* = 8 (1 detrans [1 F]) Age NS 1 med	In-depth interview and document analysis, content and thematic analysis	Conceptual description
11, Slothouber (2021), Canada, dissertation (chapter 4)	Interpret transition and detransition beyond regret narratives	*N* = 3 (3 F) Age NS 3 med	Semi-/unstructured interviews, content analysis	Thematic survey
12, *Strang et al., 2018, US, journal article	Explore the lived experience of TGD people with ASD	*N* = 22 (2 detrans [1 F, 1 M]) Age NS 2 soc^b^	Semi-structured interview, framework analysis	Thematic survey
13, Turban et al. (2021), US, journal article	Investigate reasons leading to detransition	*N* = 2242 (1235 M, 1007 F) Age range = 18–65+ 746 soc, 1496 med^b^	Survey, content analysis	Thematic survey
14, Vandenbussche (2022), Belgium, journal article	Analyze the needs of detransitioners	*N* = 237 (217 F, 20 M) *M* age = 25.02 73 soc, 164 med^b^	Survey, thematic analysis^b^	Topical survey
15, Yoo (2018), US, book chapter	Explore experiences of regret and detransition	*N* = 11 (6 detrans [4 F, 2 M], 4 TGD with regret, 1 provider) Age and transition NS	Semi-structured interview,^b^ analysis NS	Topical survey

*Notes*. F: Natal female; M: Natal male; NS: Not specified; Soc: Social transition; Med: Medical transition; TGD: Transgender and gender diverse; ASD: Autism spectrum disorders.

a Studies with the same sample.

b Inferred from the information provided in the article.

### Characteristics of included studies

Of the 15 included studies, 12 were journal articles, two were dissertations, and one was a book chapter. One study was published in 1998, while the remaining 14 were published between 2018 and 2023. The number of participants ranged from one to 2242, with a total of 2759 across all studies. However, five studies used mixed samples, so the actual number of participants who detransitioned was 2689 (*n* = ∼1350 [∼50.2 %] natal female). Of these, at least 1822 (67.8 %) had medically transitioned. Only four studies reported the mean age of participants, which ranged from 21 to 46 years.

Twelve studies were classified as qualitative. Of these, 10 used semi-structured interviews, one combined the use of in-depth interviews with document analysis, and one sampled social media posts. The remaining three studies were classified as mixed-methods, and all used closed- and open-ended survey questions. Different types of qualitative approaches were used to analyze the data, including thematic, phenomenological, content, comparative, and framework analysis. Only two studies did not specify or allow to infer the analysis technique. The findings of eight of the 15 studies were categorized as thematic surveys, five as topical surveys, and two as conceptual/thematic descriptions.

### Findings and meta-findings

The initial extraction phase yielded a total of 582 findings, which were then grouped and refined into 34 unique meta-findings with frequencies ≥ 15 %. These meta-findings are listed in Table 2.

**Table 2:** Main meta-findings.

**Themes and subthemes**	**Meta-findings**	**Frequencies**
Gender transition (T)		
T1. Perspectives	Gender dysphoria and transition desires related to previous psychological issues and vulnerabilities^3,4,5,8,9,11,14,15^	53.3 %
	Pressure to transition from friends, family, partners, or professionals^3,4,5,8,9,14,15^	46.7 %
	Lack of exploration, questioning, and understanding from healthcare professionals^3,8,9,11,15^	33.3 %
	Lack of alternatives to gender transition offered to deal with gender dysphoria^8,9,14,15^	26.7 %
	Hope that transition would bring positive changes and solutions to problems^3,4,11^	20 %
	Doubts before transitioning not expressed due to fear or pressure^3,4,11^	20 %
	Pressure to follow rigid trajectories of transition and treatment^6,11,14^	20 %
	Decision not to transition based on current knowledge and lived experiences^8,11,15^	20 %
T2. Emotions	Negative: regret, grief, guilt, shame, nostalgia, anger, anxiety, and depression^3,4,7,8,9,11^	40 %
	Ambiguous: regret, doubts, satisfaction, and appreciation of transition^2,4,7,8,11^	33.3 %
	Positive: gratitude, satisfaction, and comfort with the social/physical changes^3,7,8^	20 %
Gender detransition (D)		
D1. Driving factors	Pressure, lack of support, or discrimination^1,2,4,5,7,11,13,15^	53.3 %
	Changes, doubts, or fluctuations in gender identity and transition desires^2,4,5,12,13,14^	40 %
	Physical/emotional discomfort and/or concerns related to physical/mental health^3,7,13,14^	26.7 %
	New perspectives on the factors that contributed to gender dysphoria and transition desires or new ways of thinking about gender^3,5,8^	20 %
	Mismatch between the pre- and post-transition self, leading to feelings of inauthenticity^3,9,15^	20 %
D2. Challenges		
*a. Social and emotional difficulties*	Causing gender confusion and continuing to be perceived as TGD^6,8,9,11^	26.7 %
	Negative feelings associated with transition, difficulties in forming relationships, unresolved past psychological problems, and discomfort with physical changes^3,7,8,9^	26.7 %
*b. Lack of support and understanding*	Though some received continued support and acceptance, others experienced interpersonal questioning and rejection, leading to loneliness and alienation^2,3,6,9,11,14^	40 %
	Lack of information and resources on detransition^7,8,9,11,14^	33.3 %
*c. Negative healthcare experiences*	Negative encounters with professionals causing distrust^3,7,11,14^	26.7 %
	Stigma and lack of knowledge on detransition causing shame and disengagement from healthcare^3,7,14^	20 %
*d. Detransphobia*	Nondisclosure of detransition for fear of being judged or losing friendships^3,6,9,14^	26.7 %
	Negative stereotypes and prejudices about detransition^3,6,15^	20 %
	Questioning, invalidation, rejection, and loss of previous supports in the TGD community^3,6,14^	20 %
*e. Identity concerns*	Continued concerns about gender identity^8,9,11^	20 %
D3. Needs	Social: finding support, obtaining recognition and validation of experiences, seeing changes in the representation of gender norms^3,6,7,11,14^	23.1 %^a^
	Psychological: accepting transition and detransition, working through grief and negative feelings, obtaining support and guidance, solving previous psychological problems^3,7,14^	20 %
	Medical: receiving supervision and information on possibilities and treatments^7,11,14^	20 %
D4. Growth and evolution	Relief and happiness for not progressing further in transition, experiencing greater bodily comfort and wellbeing^3,8,9^	20 %
	Detransition as an opportunity to grow, learn, and accept oneself^3,8,10^	20 %
D5. Identity and future	Detransition has different meanings for people and does not always entail desires to reverse the changes^7,8,10,13^	26.7 %
	Some individuals retransition or consider retransitioning in the future^1,5,7,8^	26.7 %
	Detransitioners’ identities are multiple and multidimensional: some reidentify with their natal sex, sexual orientation, or as nonbinary; others move away from gender identity or understand themselves somewhere between cisgender^b^ and TGD^6,7,8,10,12^	23.1 %^a^

*Notes*. TGD: Transgender and gender diverse.

a Two studies with the same sample coincided on this meta-finding. The frequency was calculated by dividing (1) the number of studies containing this meta-finding minus the number of studies with common samples with the same meta-finding between (2) the total number of studies minus the number of studies with common samples with the same meta-finding.

b Describes people who identify with their natal sex.

The study with the highest contribution of meta-findings was Haarer (2022) (61.8 %), followed by Pullen Sansfaçon, Gelly, et al. (2023) (53 %) and Slothouber (2021) (47.5 %) (Table S4 in the Supplemental Materials). The two studies with the largest contribution of meta-findings (Haarer, 2022; Pullen Sansfaçon, Gelly, et al., 2023) reported differences between participants who had only undergone social transition and those who had also undergone medical treatment. Specifically, the former participants reported less negative feelings about their transition and expressed relief that they had not begun medical treatment. In addition, the opportunity to change their minds before making irreversible medical decisions made detransitioning easier. Of note, some studies highlighted that nonbinary gender identities may be important during detransition, either by being the primary reason for detransition (*Durwood et al., 2022; Littman, 2021), by allowing flexible gender expression (Pullen Sansfaçon, Gelly, et al., 2023), or by serving as an intermediate step before reidentifying with the natal sex (Haarer, 2022).

### Methodological quality appraisal

Overall, the methodological quality of the qualitative studies was good (Table S5 in the Supplementary Materials), meaning that their design was appropriate to answer the research questions and that the integration between data analysis and interpretation was satisfactory. One study was at moderate risk of bias (Kuiper & Cohen-Kettenis, 1998) because it was not possible to determine the method of data analysis and therefore whether the interpretation of the results was appropriate. The remaining study was at high risk of bias (Yoo, 2018) because it did not pose clearly defined research questions, did not specify the method of data analysis, and did not justify how the results were derived from the data collected.

Regarding the mixed-methods studies (Littman, 2021; Turban et al., 2021; Vandenbussche, 2022), all three were at moderate risk of bias (Table S6 in the Supplementary Materials). Although they integrated and appropriately interpreted the quantitative and qualitative components of the research, the use of mixed methods was not adequately justified, and the methodological quality criteria associated with the quantitative and qualitative traditions were not met separately.

## Discussion

This review aggregates and synthesizes qualitative data on the experiences of 2689 people who detransitioned, the slight majority natal female and most after medically transitioning, across 15 studies. Based on the 582 findings extracted, 34 meta-findings with frequencies ≥ 15 % were derived. These meta-findings were grouped into two large themes: “Gender transition,” with 11 meta-findings divided into two subthemes, and “Gender detransition,” with 23 meta-findings divided into five subthemes. Frequencies fluctuated between 20 and 53.3 %, and studies contributed between 5.9 % and 61.8 % of all meta-findings. Considering detransitioners’ diverse viewpoints on their gender process and their emotional, social, and identity struggles and needs, we aim to advance broad recommendations for psychotherapeutic work with this population.

Many detransitioners believed that their reasons for transitioning were legitimate and hoped that it would bring positive changes to their lives. However, they now think their feelings were influenced by other issues and vulnerabilities that were interpreted at the time as a sign of gender dysphoria or transness (Haarer, 2022; Littman, 2021; Pullen Sansfaçon, Gelly, et al., 2023; Slothouber, 2021). In retrospect, they wish someone had challenged them, helped clarify their motivations, and offered alternatives to transition (Pullen Sansfaçon, Gelly, et al., 2023; Sanders et al., 2023; Vandenbussche, 2022), as with their current knowledge and lived experiences they now believe transitioning was not the best decision (Pullen Sansfaçon, Gelly, et al., 2023; Slothouber, 2021; Yoo, 2018). However, some detransitioners also feel that it would have been difficult to dissuade them from transitioning or that they would have many difficulties today had they not undergone some of the medical procedures associated with their transition (Pullen Sansfaçon, Gelly, et al., 2023; Slothouber, 2021). Others are grateful for the opportunity to transition and express satisfaction and comfort with their physical changes (Haarer, 2022; MacKinnon, et al., 2022b; Pullen Sansfaçon, Gelly, et al., 2023). Therefore, detransitioners represent a heterogeneous population in terms of their experiences and perspectives about their gender transition process.

When transition involves irreversible medical treatments, permanent physical changes can become a continuous reminder of the decisions made and thus complicate the grieving process (e.g., Gribble et al., 2023). The nostalgia for the pre-transition self and its perceived conflict with the post-transition self may be a painful and recurrent experience. Social unintelligibility (e.g., continuing to be perceived as TGD; Haarer, 2022; MacKinnon et al., 2022a; Pullen Sansfaçon, Gelly, et al., 2023; Sanders et al., 2023; Slothouber, 2021), difficulties in belonging and establishing relationships post-detransition (Sanders et al., 2023), and overall stigma, lack of support, information, and sympathy across social and healthcare contexts (Haarer, 2022; MacKinnon et al., 2022a, MacKinnon et al., 2022b; Pullen Sansfaçon, Gelly, et al., 2023; Sanders et al., 2023; Slothouber, 2021; Vandenbussche, 2022) can at the same time increase and downplay the grief that may come with medically detransitioning. Furthermore, some detransitioners may feel concerned about the possibility of experiencing doubts about their gender identity again (Pullen Sansfaçon, Gelly, et al., 2023; Sanders et al., 2023; Slothouber, 2021). This persistent uncertainty can make detransitioners feel incapable of achieving stability or living a fulfilling life.

Providers should acknowledge feelings of loss and grief and validate detransitioners’ experiences, perspectives, and meaning making. This includes providing guidance and support to identify and express emotions, both positive and negative, as well as ambiguity and contradiction, creating therapeutic space for complexity. It also involves recognizing and accepting the idea that life may never be the same again and that ambiguity may never be fully resolved. Detransition may also reveal or resurface past psychological problems and vulnerabilities, or bring new emotional difficulties (Haarer, 2022; Pullen Sansfaçon, Gelly, et al., 2023; Sanders et al., 2023). In some cases, these will be related to unresolved mental health issues, such as gender dysphoria, depression, anxiety, or eating and body image problems. In others, they will be related to unresolved traumatic events, distorted beliefs (e.g., gender rigidity, internalized misogyny/homophobia), or new forms of bodily discomfort (e.g., “reverse” gender dysphoria) with important implications for detransitioners’ mental health. Thus, it will be crucial for mental health professionals to provide adequate and ongoing psychological treatment and support tailored to each detransitioner's circumstances, pending evidence on what specific approaches might be more effective and helpful in this endeavor.

At the same time, however, it is important to consider ways of moving forward. On the one hand, acknowledging loss, grief, and other negative emotions does not preclude recognizing other aspects of detransitioners’ lives that have not been lost, which can help them to focus on their personal strengths and new opportunities. This fosters resilience and can help detransitioners make sense of their journey and discover positive aspects and learnings, both about themselves (e.g., freedom from gender norms and expectations) and about the world (e.g., more flexible notions of gender) (see Haarer, 2022; Pullen Sansfaçon, Gelly, et al., 2023). Additionally, recognizing the desire for positive change that underlies gender transition (e.g., Haarer, 2022) and reframing it as part of the universal human search for meaning and wellbeing can be a useful strategy to alleviate detransitioners’ feelings of disconnection and estrangement. On the other hand, providing information, resources, informal support, and fostering connections with other detransitioners can be important in the recovery process (Haarer, 2022; MacKinnon et al., 2022a, MacKinnon et al., 2022b; Slothouber, 2021; Vandenbussche, 2022). In this regard, associations and social networks can provide a space to share experiences, receive support, and address social alienation, although they are not always sufficient to meet the needs of detransitioners (MacKinnon et al., 2022a; Sanders et al., 2023).

In summary, psychotherapeutic work with detransitioners must aim to recognize their experiences and emotions, validate ambiguity, find meaning, and discover new possibilities for the future, while addressing the negative aspects that detransitioners may already be experiencing in their daily lives (e.g., invalidation, misunderstanding, prejudice, etc.) or that have been problematic for them in the past (e.g., gender rigidity and stereotypes). Mental health professionals should strive to tolerate and give space to complex and contradictory emotions, and to approach detransition with curiosity, openness, sensitivity, and respect. In this way, providers can increase their understanding of the phenomenon and become figures of support and trust for detransitioners seeking help. This can be a starting point to address issues such as lack of information, understanding, and support; stigma, disengagement from healthcare, and unmet mental health needs (Haarer, 2022; MacKinnon et al., 2022a, MacKinnon et al., 2022b; Sanders et al., 2023; Slothouber, 2021; Vandenbussche, 2022).

While this is particularly relevant for detransition experiences that involve negative emotions and ambiguity, which is a challenging scenario for mental health professionals, some detransitioners feel grateful, satisfied, and comfortable with their physical changes, and appreciate and recognize the opportunity to transition despite the difficulties (Haarer, 2022; MacKinnon et al., 2022b; Pullen Sansfaçon, Gelly, et al., 2023). Therefore, it is important to be aware of this diversity in detransition experiences. This also means avoiding the assumption that all detransitioners wish to return to a pre-transition state, or that they will reidentify with their natal sex. In this context, it is important to remember that detransition does not mean the same to everyone and that not everyone wants to reverse the changes experienced during transition (MacKinnon et al., 2022b; Pullen Sansfaçon, Gelly, et al., 2023; Turban et al., 2021). Moreover, post-detransition experiences and identities are multiple and multidimensional (MacKinnon, Gould, et al., 2022a, MacKinnon et al., 2022b; Pullen Sansfaçon, Gelly, et al., 2023), and some people, particularly those who detransition for external reasons (e.g., pressure, lack of support), leave open the possibility of retransitioning in the future (Cain & Velasco, 2021; Littman, 2021; MacKinnon et al., 2022b; Pullen Sansfaçon, Gelly, et al., 2023). Thus, transition and detransition pathways are not always linear and premature identity foreclosure should be avoided.

Finally, this review may also be useful in providing support and guidance to individuals considering detransition. The process of exploration and decision making that leads to detransition resembles the process that precedes gender transition (MacKinnon et al., 2022a), which is marked by significant discomfort and uncertainty caused by the lack of information (Sanders et al., 2023). Detransition is a viable, dignified, and livable option, so it is essential to create space for people to explore it freely and without prejudice. Otherwise, we risk fostering detransphobia by conveying a distorted image of detransitioners’ lives as unlivable and hopeless, and encouraging disengagement from healthcare leading some people to navigate detransition alone without support, resources, or information (e.g., Haarer, 2022; MacKinnon et al., 2022b). Yet, it is important that the provision of support for potential detransition is not based on invalidating TGD identities or relief at discontinuing transition, as this could be perceived negatively by some detransitioners (*Durwood et al., 2022; MacKinnon et al., 2022a).

In terms of limitations of this review, we excluded meta-findings that did not exceed the 15 % threshold. It should be noted that frequencies convey prevalence, not value. Therefore, it is likely that some of these meta-findings would have provided new nuances or perspectives for understanding detransition. Second, we included three studies with mixed (*Durwood et al., 2022; Yoo, 2018) or partially mixed (Sanders et al., 2023) samples, so their findings should be taken with caution. Similarly, the study with the largest sample (Turban et al., 2021) included participants who identified as TGD at the time of data collection, whose experiences may not be generalizable to the broader population of detransitioners. Thirdly, two of the three mixed methods studies (Littman, 2021; Vandenbussche, 2022) were categorized as such based on their methodological similarity to Turban et al.’s (2021) study, but it could be argued whether their method of data collection and analysis is sufficient for this categorization.

In terms of strengths, we first note the inclusion of studies with research objectives not specifically related to detransition (Shepherd & Hanckel, 2021; *Strang et al., 2018), which provided additional information that would have gone unnoticed had the search been limited to detransition-focused articles. Second, we calculated frequencies for each meta-finding, which provided further information and meaning (Sandelowski & Barroso, 2007). In addition, only meta-findings with frequencies ≥ 15 % were included, that is, present in at least three of the 15 included studies. Thus, the 34 meta-findings represent a feasible set of findings that capture the essence of detransition. Finally, the inclusion of dissertations resulted in a large contribution of meta-findings.

## Conclusions

This article presents a systematic review and metasummary of studies with qualitative data on the experiences of people who detransition. After conducting an exhaustive search, we included 15 studies with 2689 participants who detransitioned, yielding 34 meta-findings with frequencies ≥ 15 %. Altogether, these revealed important insights for understanding and developing broad recommendations for psychotherapeutic work with detransitioners.

Findings highlight that detransitioners are diverse in terms of the reasons leading to detransition and the perspectives and emotions associated with their initial transition. Some feel that it was a mistake and experience regret, grief, or anger, wishing that someone had supported them in clarifying their motivations or understanding that their gender-related distress was rooted in other issues and vulnerabilities. Despite the difficulties, other detransitioners feel satisfied and grateful for the opportunity to transition and recognize positive aspects, such as evolution, growth, and learning, while others experience conflicting and ambiguous feelings. Mental health professionals should be aware of this diversity of experiences and perspectives when working with detransitioners.

Often, detransition is associated with significant challenges, such as lack of social and professional support, information, and resources; interpersonal difficulties, mental health issues, identity concerns, and detransphobia, characterized by stigma, loss of previous supports, and prejudices around detransition. These result in unmet psychological, social, and medical needs. A psychotherapeutic approach focused on the recognition, validation, and expression of complex emotions will serve as a basis for identity reconstruction, search for meaning, and discovery of personal strengths and possibilities. Additional tailored treatment of concurrent pre- or post-detransition psychological issues, along with the promotion of social connections and resources, will provide a foundation to increase detransitioners’ wellbeing and sense of belonging. However, detransition does not necessarily conclude TGD identity and expression. Continued efforts by mental health providers are essential to support decision making, identity exploration, and healthcare engagement, thus ensuring that the lives of detransitioners are as dignified, fulfilling, and satisfactory as possible.

## Funding

This article has received Open Access funding from the Osaklinik research group (IT-1450-22) of the Government of the Basque Country.

## Declaration of competing interest

The authors declare that they have no potential conflicts of interest.

## Appendix Supplementary materials

Image, application 1
