# Supplementary material for: A qualitative metasummary of detransition experiences with recommendations for psychological support
Source: Int J Clin Health Psychol. 2024 May 7;24(2):100467. doi: 10.1016/j.ijchp.2024.100467 (PMC11091498; doi:10.1016/j.ijchp.2024.100467)
Supplement: Supplementary file 1 [file mmc1.docx]

**Table S1.** Search strategy

| 1 | detransition* OR de-transition* OR retransition* OR re-transition* OR reidentif* OR re-identif* OR regret* OR discontinu* OR terminat* OR rever* OR desist* OR disidentif* OR dis-identif* |
| --- | --- |
| 2 | qualitative* OR “qualitative data” OR “qualitative stud*” OR “qualitative method*” OR “qualitative research” OR “qualitative report” OR survey OR interview OR “mixed method*” OR “case stud*” OR “case report*” OR vignette* OR “clinical scenario*” OR “composite case*” OR “focus group*” OR “exploratory study” OR “descriptive study” OR “thematic analysis” OR “grounded theory” OR “content analysis” OR “narrative analysis” OR “discourse analysis” OR phenomenolog* OR hermeneutic* OR interpretive OR ethnograph* OR ethnometodolog* OR “participant observation” OR observation |
| 3 | “gender identity” OR “gender presentation” OR “gender expression” OR “gender role” OR “gender trajector*” OR “gender dysphoria” OR “gender incongruence” OR transgender OR nonbinary OR non-binary OR “gender divers*” OR “gender creativ*” OR “gender expansiv*” OR “gender nonconforming” OR “gender non-conforming” OR “gender minorit*” OR genderqueer OR agender OR transsexual* OR “gender affirm*” |
| 4 | “human immunodeficiency virus” OR HIV OR “acquired immunodeficiency syndrome” OR AIS OR “sexually transmitted infection*” OR STI* OR “sexually transmitted disease*” OR STD* OR “pre-exposure prophylaxis” OR PrEP |
| 5 | #1 AND #2 AND #3 NOT #4 |

*Notes*. We used truncation with asterisks to expand the search terms according to their lexical family. After the preliminary search, we refined the terms in row 1, added quotation marks, used the NOT Boolean operator (row 4), and applied language filters to reduce the number of results not relevant to the review.

**Table S2.** Inclusion and exclusion criteria according to the SPIDER tool

| **Criteria** | **Inclusion** | **Exclusion** |
| --- | --- | --- |
| Sample (S) | Individuals who stop or reverse the social, medical, and/or legal aspects of their gender transition, regardless of their age, how they currently conceptualize their gender identity, or the steps they have taken to detransition | Individuals who have not taken the steps commonly understood to be part of a gender transition; individuals who have not detransitioned or are considering detransition but have not detransitioned (exclusively) |
| Phenomenon of Interest (PI) | Gender detransition | Gender transition; retransition (resuming a gender transition); desistance (remission of gender dysphoria prior to social or medical transition); reversal of assigned sex in individuals with disorders of sexual development; reidentification with the natal sex in the context of dementia |
| Design (D) | Any type of data collection method that can provide qualitative information about detransition experiences | Quantitative data collection methods |
| Evaluation (E) | Psychosocial experiences and challenges associated with detransition, including: factors (internal/external) influencing the decision to detransition; social and emotional experiences (positive/negative) associated with detransition; barriers and facilitators to seeking professional support; and reflections on transition and detransition | Any other aspect not related to the experience of detransition |
| Research type (R) | Qualitative and mixed methods with separate analysis and presentation of qualitative data (including dissertations and book chapters) | Quantitative and mixed methods without separate analysis and presentation of qualitative data; systematic reviews, theoretical and opinion studies, abstracts/conferences/other types of grey literature, autobiographical accounts |

**Table S3.** Typology of qualitative findings according to Sandelowski and Barroso (2007)

| **Type of qualitative finding** | **Description** |
| --- | --- |
| No finding | Presentation of uninterpreted data as if they were findings |
| Topical survey | Organization of data in lists or inventories of topics covered by participants |
| Thematic survey | Description of patterned responses (themes) in the topics brought up by participants |
| Conceptual/thematic description | Interpretive use of concepts or themes to reframe a phenomenon or experience |
| Interpretative explanations | Development of coherent models that address causality or the nature of a phenomenon or experience |

*Notes*. Types of qualitative findings are organized from lowest (top) to highest (bottom) degree of transformation and interpretation of the data by the authors.

**Table S4.** Contribution of included studies (from highest to lowest)

| **Studies** | **Contribution (number of meta-findings)** |
| --- | --- |
| Haarer (2022) | 61.8% (21/34) |
| Pullen Sansfaçon, Gelly, et al. (2023) | 53% (18/34) |
| Slothouber (2021) | 47.5% (16/34) |
| MacKinnon, Kia, et al. (2022) | 44.1% (15/34) |
| Vandenbussche (2022) | 44.1% (15/34) |
| Sanders et al. (2023) | 38.2% (13/34) |
| Kuiper & Cohen-Kettenis (1998) | 23.5% (8/34) |
| MacKinnon, Gould, et al. (2022) | 23.5% (8/34) |
| Yoo (2018) | 23.5% (8/34) |
| Littman (2021) | 17.6% (6/34) |
| Durwood et al. (2022) | 11.8% (4/34) |
| Turban et al. (2021) | 11.8% (4/34) |
| Shepherd & Hanckel (2021) | 8.8% (3/34) |
| Cain & Velasco (2021) | 5.9% (2/34) |
| Strang et al. (2018) | 5.9% (2/34) |

**Table S5**. Appraisal of qualitative studies using the MMAT

| **Methodological quality criteria** | **1** | **2** | **3** | **4** | **5** | **6** | **7** | **8** | **9** | **10** | **11** | **12** |
| --- | --- | --- | --- | --- | --- | --- | --- | --- | --- | --- | --- | --- |
| 1. Are there clear research questions? | Y | Y | Y | Y | Y | Y | Y | Y | Y | Y | Y | N |
| 1. Do the collected data allow to address the research questions? | Y | Y | Y | Y | Y | Y | Y | Y | Y | Y | Y | CT |
| 1. Is the qualitative approach appropriate to answer the research question? | Y | Y | Y | Y | Y | Y | Y | Y | Y | Y | Y | CT |
| 1. Are the qualitative data collection methods adequate to address the research question? | Y | Y | Y | Y | Y | Y | Y | Y | Y | Y | Y | CT |
| 1. Are the findings adequately derived from the data? | Y | Y | Y | CT | Y | Y | Y | Y | Y | Y | Y | CT |
| 1. Is the interpretation of results sufficiently substantiated by the data? | Y | Y | Y | CT | Y | Y | Y | Y | Y | Y | Y | CT |
| 1. Is there coherence between qualitative data sources, collection, analysis and interpretation? | Y | Y | Y | CT | Y | Y | Y | Y | Y | Y | Y | CT |

*Notes*. Y: Yes; N: No; CT: Can’t tell. Study references: 1 = Cain and Velasco (2021); 2 = Durwood et al. (2022); 3 = Haarer (2022); 4 = Kuiper & Cohen-Kettenis (1998); 5 = MacKinnon, Gould, et al. (2022); 6 = MacKinnon, Kia, et al. (2022); 7 = Pullen Sansfaçon, Gelly, et al. (2023); 8 = Sanders et al. (2023); 9 = Shepherd & Hanckel (2021); 10 = Slothouber (2021); 11 = Strang et al. (2018); 12 = Yoo (2018).

**Table S6**. Appraisal of mixed methods studies using the MMAT

| **Methodological quality criteria** | **13** | **14** | **15** |
| --- | --- | --- | --- |
| 1. Are there clear research questions? | Y | Y | Y |
| 1. Do the collected data allow to address the research questions? | Y | Y | Y |
| 1. Is there an adequate rationale for using a mixed methods design to address the research question? | N | N | N |
| 1. Are the different components of the study effectively integrated to answer the research question? | Y | Y | Y |
| 1. Are the outputs of the integration of qualitative and quantitative components adequately interpreted? | Y | Y | Y |
| 1. Are divergences and inconsistencies between quantitative and qualitative results adequately addressed? | CT | CT | CT |
| 1. Do the different components of the study adhere to the quality criteria of each tradition of the methods involved? | N | N | N |

*Notes*. Y: Yes; N: No; CT: Can’t tell. Study references: 13 = Littman (2021); 14 = Turban et al. (2022); 15 = Vandenbussche (2022).
